# HLA and non-HLA genes in Behçet’s disease: a multicentric study in the Spanish population

**DOI:** 10.1186/ar4328

**Published:** 2013-10-04

**Authors:** Marco Antonio Montes-Cano, Marta Conde-Jaldón, José Raul García-Lozano, Lourdes Ortiz-Fernández, Norberto Ortego-Centeno, María Jesús Castillo-Palma, Gerard Espinosa, Genaro Graña-Gil, Miguel Angel González-Gay, Ana Celia Barnosi-Marín, Roser Solans, Patricia Fanlo, Teresa Camps, Santos Castañeda, Juan Sánchez-Bursón, Antonio Núñez-Roldán, Javier Martín, María Francisca González-Escribano

**Affiliations:** 1 Servicio de Inmunología, IBiS, Hospital Universitario Virgen del Rocío/CSIC/Universidad de Sevilla, Sevilla, Spain; 2 Servicio de Medicina Interna, Hospital Clínico San Cecilio, Granada, Spain; 3 Servicio de Medicina Interna, Hospital Universitario Virgen del Rocío, Sevilla, Spain; 4 Servicio de Enfermedades Autoinmunes, Hospital Clinic, Barcelona, Spain; 5 Servicio de Reumatología, CHU A Coruña, Coruña, Spain; 6 Servicio de Reumatología, Hospital Marques de Valdecilla, Santander, Spain; 7 Servicio de Medicina Interna, Hospital Torrecárdenas, Almería, Spain; 8 Servicio de Medicina Interna, Hospital Vall d’Hebron, Barcelona, Spain; 9 Servicio de Medicina Interna, Hospital Virgen del Camino, Pamplona, Spain; 10 Servicio de Medicina Interna, Hospital Carlos Haya, Málaga, Spain; 11 Servicio de Reumatología, Hospital de la Princesa, Madrid, Spain; 12 Servicio de Reumatología, Hospital de Valme, Sevilla, Spain; 13 IPB López Neyra, Granada, Spain

## Abstract

**Introduction:**

According to genome wide association (GWA) studies as well as candidate gene approaches, Behçet’s disease (BD) is associated with human leukocyte antigen (HLA)-A and HLA-B gene regions. The HLA-B51 has been consistently associated with the disease, but the role of other HLA class I molecules remains controversial. Recently, variants in non-HLA genes have also been associated with BD. The aims of this study were to further investigate the influence of the HLA region in BD and to explore the relationship with non-HLA genes recently described to be associated in other populations.

**Methods:**

This study included 304 BD patients and 313 ethnically matched controls. HLA-A and HLA-B low resolution typing was carried out by PCR-SSOP Luminex. Eleven tag single nucleotide polymorphisms (SNPs) located outside of the HLA-region, previously described associated with the disease in GWA studies and having a minor allele frequency in Caucasians greater than 0.15 were genotyped using TaqMan assays. Phenotypic and genotypic frequencies were estimated by direct counting and distributions were compared using the χ^2^ test.

**Results:**

In addition to HLA-B*51, HLA-B*57 was found as a risk factor in BD, whereas, B*35 was found to be protective. Other HLA-A and B specificities were suggestive of association with the disease as risk (A*02 and A*24) or protective factors (A*03 and B*58). Regarding the non-HLA genes, the three SNPs located in *IL23R* and one of the SNPs in *IL10* were found to be significantly associated with susceptibility to BD in our population.

**Conclusion:**

Different HLA specificities are associated with Behçet’s disease in addition to B*51. Other non-HLA genes, such as *IL23R* and *IL-10*, play a role in the susceptibility to the disease.

## Introduction

Behçet’s disease (BD) is a systemic vasculitis characterized mainly by recurrent oral and genital ulceration, although other clinical manifestations, such as skin lesions, ocular, gastrointestinal and neurological disorders, are relatively common [1]. The aetiology of BD remains unclear; nevertheless, imbalances in the innate or adaptive immune response triggered by infectious agents or environmental factors in genetically predisposed individuals could be some of the underlying mechanisms of the disease. Evidence of genetic contributions to the pathogenesis of the disease are based on familial aggregation, predominance in patients with Mediterranean or Asian ancestry and association with the human leukocyte antigen (HLA) B51 (HLA-B51) region in several ethnic groups. Multiple studies investigating associations between HLA and BD have been reported [2,3]. All of them described an association between the disease and the specificity HLA-B51 independent of the ethnicity of the study population [4]. Nevertheless, associations of this pathology with other HLA-B allele groups are less consistent [5-12]. This lack of replication may be due to the lack or weaker association of BD with other HLA-B allele groups, as well as with the relatively small sample size included in each study, which makes it difficult to find an association with relatively underrepresented allele groups. The typing procedures have evolved to achieve a better resolution. Nevertheless, the basis of the association of HLA with BD remains elusive. Very recently, in a study employing dense genotyping in the HLA region, three peaks of association with the disease were found: one of these single-nucleotide polymorphisms (SNPs) is located in the HLA-A region, another is located in the HLA-B human major histocompatibility complex class I chain-related gene A (HLA-B-MICA) region and another is located in the HLA-C region. After inferring the classical HLA alleles from the SNPs, the authors of that suggested that the robust association between HLA-B*51 and BD is explained by a variant located between the *HLA-B* and *MICA* genes [13]. Improved knowledge of the HLA-B molecules associated with BD would help to establish the basis of this association. Contribution of the HLA region has been estimated to represent approximately 20% of the genetic component of this disease [14]. Different studies designed to establish the contribution of other HLA and non-HLA genes have been published. Two different approaches have been used in genetic association studies and in BD. First, the candidate gene strategy has permitted the establishment of the association of BD with HLA-B51 and with other HLA and non-HLA genes [15]. Second, the free hypothesis approach has also been used to assess the genetic component of BD [16-20]. Three of these studies, which were carried out using a large-scale SNP genotyping strategy (genomewide association studies (GWASs)), were recently published [18-20]. In general, these studies confirmed the association with HLA-B, identified a second HLA class I region independently associated with the disease in the HLA-A region and described an association with other non-HLA regions [19,20]. The two aims of the present study were to further investigate the influence of the HLA region in BD and to explore the relationship with the previously described non-HLA genes associated with BD.

## Methods

A total of 304 BD patients (134 males and 170 females) with a mean age at onset ± SD of 38.7 ± 13.8 years fulfilled the 1990 International Study Group classification criteria for BD [21] and they were recruited into the study. A total of 313 bone marrow and blood donors (50% males) were included in the study as normal healthy controls. All the participants were Spanish Europeans recruited from different Spanish hospitals. The study was approved by all local ethical committees of the corresponding hospitals, and all the study participants gave their written informed consent to participate. Clinical features of the patient group were as follows: 100% had oral ulcers; 74.4% had genital ulcers; 49.8% had uveitis; 47.4% had arthritis; and 24.5% had vascular involvement, 18.1% had neurological involvement and 19.1% had gastrointestinal. The distribution of the frequencies of the different markers in the cohorts from different hospitals was not significantly different.

Peripheral blood was obtained from the healthy controls, and peripheral blood or saliva served as the starting material obtained from patients. Samples were obtained from participants after they provided their written informed consent. Genomic DNA was extracted using the QIAamp DNA Mini Kit (Qiagen, Barcelona, Spain) according to the manufacturer’s recommendations and stored at -20°C. The purity of DNA was determined using a NanoDrop spectrophotometer (Thermo Fisher Scientific, Wilmington, DE, USA). Only DNA samples having a 260 nm to 280 nm absorbance ratio between 1.7 and 2.0 and a final concentration of 10 to 20 ng/μl were considered appropriate. Ten DNA samples from saliva were eliminated because they did not meet these quality criteria.

HLA-A and HLA-B low-resolution typing was carried out by using the polymerase chain reaction sequence-specific oligonucleotide (PCR-SSOP) Luminex method using LABType SSO (One Lambda Inc., Canoga Park, CA, USA) according to the manufacturer’s instructions. Briefly, target DNA was PCR-amplified using HLA-A or HLA-B group-specific primers. The biotinylated PCR products were denatured and hybridized with specific probes bound to color-coded microspheres. Phycoerythrin-conjugated streptavidin was used to label and reveal reactions, and a flow analyser, LABScan 100 (One Lambda Inc), was used to identify fluorescence intensity in each microsphere. The software HLA Fusion 2.0 (One Lambda Inc) was used to assign the HLA typing of each locus. Typing of locus A and locus B was possible for 92% and 93%, respectively, of the DNA samples meeting the quality criteria.

Additionally, an intergene (*LOC100129342*) and six gene regions (*IL23R*, *IL10*, *CPVL*, *TDRD7/C9orf174*, *UBASH3B* and *UBAC2*) previously reported to be associated with BD in GWASs in candidate gene studies were included [18-20,22]. The SNPs were selected from among those included in previous publications, taking into account the minor allele frequency (MAF) in the Utah residents with Northern and Western European ancestry from the Centre d’Etude du Polymorphisme Humain collection (CEU) population [23]. Only SNPs with MAF greater than 0.15 in the CEU were included in this study, one each for *LOC100129342* (rs11206377), *CPVL* (rs317711), *TDRD7* (rs2061634), *UBASH3B* (rs4936742) and *UBAC2* (rs7999348) and three for each of the two non-HLA loci with the highest evidence of association with BD in different populations, which are *IL23R* (rs17375018, rs7517847 and rs1343151) and *IL10* (rs3024498, rs2222202 and rs1800872). Genotyping of these SNPs was performed using the TaqMan SNP Genotyping Assay (Applied Biosystems, Barcelona, Spain) and the LightCycler 480 System (Roche, Barcelona, Spain).

Phenotypic and genotypic frequencies were estimated by direct counting, and distributions were compared using the χ^2^ test. Odds ratios (ORs) and 95% confidence intervals (95% CI) were calculated according to Woolf’s method using the StatCalc calculator with the EPI Info software package (Centers for Disease Control and Prevention, Atlanta, GA, USA). In the case of the HLA univariate analysis, *P*-values ≤0.001 (0.05 divided by 48 which is the number of groups is the number of HLA alleles found in our population) were considered significant, whereas *P*-values between 0.01 and 0.001 were considered to be suggestive of an association and *P*-values between 0.05 and 0.01 were considered marginally associated. The adjusted ORs were calculated after constructing a logistic regression model categorizing all the individuals according to the presence or absence of the HLA-A and HLA-B groups with *P* < 0.05 in the univariate analysis (Epi Info 2002). In the cases of HLA risk factors, the analysis proposed by Svejgaard and Ryder to detect the strongest HLA association was also used. This analysis consisted of different two-tailed Fisher’s exact tests performed in various 2 × 2 tables using the GraphPad Software QuickCalcs tool (http://graphpad.com/quickcalcs/contingency1.cfm; GraphPad Software, La Jolla, CA, USA), starting with the basic data collected in a 2 × 4 table that included the four phenotypic combinations of the two HLA factors under study [24]. The ORs were calculated according to Haldane’s modification of Woolf’s method, and *P*-values of tests 1 through 4 were corrected by a factor of 6 as these authors suggested.

Gene–gene interactions were evaluated using the nonparametric multifactor dimensionality reduction (MDR) method [25-27]. A tenfold cross-validation was used to estimate the balanced testing accuracy (TA) and cross-validation consistency (CVC) of MDR models. Balanced accuracy is (sensitivity + specificity)/2, where sensitivity is true-positive/(true-positive + false-negative) and specificity is true-negative/(false-positive + true-negative). The MDR model with the highest TA and CVC values was selected as the best model for studying from one to *n* loci. Statistical significance was evaluated using a 10,000-fold permutation test.

## Results

Table 1 displays allele frequencies of the HLA-A and HLA-B groups in Spanish BD patients and healthy controls. The statistical power ranked from 8 to 40 for detecting ORs ≥1.2. Distribution of the frequencies of the 19 HLA-A alleles that were found in our population had a statistically significant difference in patients and controls (*P* = 0.005 in a 2 × 19 contingency table). Three of the HLA-A alleles were suggestive of an association with susceptibility to BD. Two of these HLA-A alleles had a higher frequency in patients: A*02 (0.34 vs. 0.26, *P* = 0.003, OR = 1.47, 95% CI = 1.14 to 1.91) and A*24 (0.11 vs. 0.07, P = 0.01, OR = 1.70, 95% CI = 1.10 to 2.52). One allele, HLA-A*03, had a lower frequency in patients (0.06 vs. 0.11, *P* = 0.003, OR = 0.53, 95% CI = 0.34 to 0.83). With respect to HLA-B alleles, distribution of the 29 HLA-B specificities found in our population was statistically significantly different in patients and controls (*P* < 10^–5^ in a 2 × 29 contingency table). In addition to the association repeatedly described with B*51 (0.23 vs. 0.07, *P* < 10^–7^, OR = 4.11, 95% CI = 2.79 to 6.06), two other HLA-B alleles were associated with BD. One of them, B*57, had a higher frequency among patients (0.06 vs. 0.02, *P* = 0.00008, OR = 3.70, 95% CI = 1.78 to 7.83), whereas the other, B*35, had a lower frequency in patients (0.07 vs. 0.12, *P* = 0.0007, OR = 0.49, 95% CI = 0.32 to 0.76). Moreover, HLA-B*58 was suggestive of an association as protective (0.002 vs. 0.02, *P* = 0.007, OR = 0.10, 95% CI = 0.0 to 0.71) and HLA-B*18 (0.05 vs. 0.09, *P* = 0.02, OR = 0.58, 95% CI = 0.35 to 0.95) and B*38 (0.02 vs. 0.04, *P* = 0.03, OR = 0.48, 95% CI = 0.23 to 0.99) had a marginal association also as protective. We categorized all the individuals according to the presence or absence of HLA with *P* < 0.05 in the univariate analysis and calculated the adjusted ORs using a logistic regression model (Table 2). According to this analysis, the ORs conferred by HLA-A*24, B*35, B*51, B*57 and B*58 remained significant and similar after adjustment.

**Table 1:** **Allelic frequencies of the HLA-A and HLA-B allele groups in Behçet’s disease patients and healthy controls**^**a**^

**Region**	**BD patients**	**Controls**	***P***	**OR (95% CI)**
HLA-A	2*n* = 542 (%)	2*n* = 626 (%)		
01	51 (0.09)	63 (0.10)		
02	186 (0.34)	164 (0.26)	0.003	1.47 (1.14 to 1.91)
03	34 (0.06)	70 (0.11)	0.003	0.53 (0.34 to 0.83)
11	31 (0.06)	47 (0.08)		
23	20 (0.04)	25 (0.04)		
24	59 (0.11)	42 (0.07)	0.01	1.70 (1.10 to 2.62)
25	3 (0.005)	8 (0.01)		
26	19 (0.03)	27 (0.04)		
29	40 (0.07)	50 (0.08)		
30	23 (0.04)	33 (0.05)		
31	19 (0.03)	13 (0.02)		
32	18 (0.03)	22 (0.03)		
33	11 (0.02)	20 (0.03)		
34	2 (0.004)	0 (0.0)		
36	1 (0.002)	0 (0.0)		
66	7 (0.01)	5 (0.008)		
68	16 (0.03)	32 (0.05)		
69	1 (0.002)	4 (0.006)		
80	1 (0.002)	1 (0.002)		
HLA-B	2*n* = 548 (%)	2*n* = 626 (%)		
07	34 (0.06)	40 (0.06)		
08	25 (0.05)	25 (0.04)		
13	5 (0.009)	5 (0.008)		
14	35 (0.06)	57 (0.09)		
15	35 (0.06)	24 (0.04)		
18	29 (0.05)	55 (0.09)	0.02	0.58 (0.35 to 0.95)
27	16 (0.03)	19 (0.03)		
35	36 (0.07)	78 (0.12)	0.0007	0.49 (0.32 to 0.76)
37	3 (0.005)	3 (0.004)		
38	12 (0.02)	28 (0.04)	0.03	0.48 (0.23 to 0.99)
39	5 (0.009)	10 (0.02)		
40	14 (0.02)	22 (0.03)		
41	5 (0.009)	7 (0.01)		
42	1 (0.002)	0 (0.0)		
44	70 (0.13)	88 (0.14)		
45	8 (0.01)	16 (0.03)		
46	1 (0.002)	0 (0.0)		
47	1 (0.002)	1 (0.002)		
48	0 (0.0)	2 (0.003)		
49	17 (0.03)	35 (0.06)		
50	13 (0.02)	20 (0.03)		
51	125 (0.23)	42 (0.07)	<10^–7^	4.11 (2.79 to 6.06)
52	5 (0.009)	8 (0.01)		
53	8 (0.01)	14 (0.02)		
54	0 (0.0)	1 (0.002)		
55	6 (0.01)	3 (0.005)		
56	4 (0.007)	1 (0.002)		
57	34 (0.06)	11 (0.02)	0.00008	3.70 (1.78 to 7.83)
58	1 (0.002)	11 (0.02)	0.007	0.10 ( 0.0 to 0.71)

^a^BD, Behçet’s disease; CI, confidence interval; OR, odds ratio; HLA, human leukocyte antigen. An allelic model was used. Allelic frequencies, calculated as the number of each specific allele divided by the total number of alleles (number of individuals × 2 (2*n*)), are shown within parentheses. Only those *P*-values ≤0.05 are indicated. P-values ≤0.001 were considered statistically significant, and the corresponding HLAs were considered to be associated. HLAs with *P*-values between 0.001 and 0.01 were considered suggestive of association, and those with *P*-values between 0.01 and 0.05 were considered marginally associated. The rest of the HLA specificities described (A*43, A*74, B*59, B67, B*73, B*78, B*81 and B*83) were not detected in our population.

**Table 2:** **Logistic regression analysis**^**a**^

**HLA**	***P***	**OR adjusted values (95% CI)**
A*02	0.40	1.18 (0.80 to 1.72)
A*03	0.11	0.66 (0.38 to 1.11)
A*24	0.02	1.84 (1.11 to 3.04)
B*18	0.06	0.59 (0.35 to 1.02)
B*35	0.008	0.50 (0.30 to 0.84)
B*38	0.20	0.61 (0.29 to 1.30)
B*51	<10^–5^	4.96 (3.21 to 7.66)
B*57	0.0002	4.16 (1.98 to 8.76)
B*58	0.04	0.11 (0.01 to 0.91)

^a^CI, confidence interval; HLA, human leukocyte antigen; OR, odds ratio. The model included those HLA factors with *P* < 0.05 in the univariate analysis.

To further clarify whether the association of the different HLA risk factors detected in the univariate analysis were independent of HLA-B*51, we performed the analysis proposed by Svejgaard and Ryder to detect the strongest HLA association. Different two by four tables were built with the phenotypic combinations of HLA-B*51 and HLA-A*02, HLA-A*24 and HLA-B*57 (Table 3). In the cases of HLA-A*24 and HLA-B*57 the ORs were greater than 1 in both HLA-B*51 positive (test 1) and negative (test 2) individuals, although only in the second case the test reached statistical significance. Nevertheless, these tests 1 and 2 were not significant for HLA-A*02. The combined associations of HLA-A*24 and also of B*57 with HLA-B*51 increased the individual risks, but this was not the case of A*02 (test 3). Finally, the contribution of HLA-A*24 and HLA-B*57 were similar to the contribution of B*51 whereas the contribution of A*02 was significantly different (test 4).

**Table 3:** **Basic data and 2 × 2 comparisons to detect the strongest human leukocyte antigen association of the risk factors**^**a**^

A*02	B*51		**BD patients**		**Controls**	
+	+		72		29	
+	–		76		112	
–	+		45		11	
–	–		73		161	
A*24	B*51					
+	+		26		6	
+	–		36		30	
–	+		91		34	
–	–		119		237	
B*57	B*51					
+	+		8		0	
+	–		24		11	
–	+		111		40	
–	–		131		262	
Factor 1-B*51	A*02		A*24		B*57	
	***P***_**c**_	**OR**	***P***_**c**_	**OR**	***P***_**c**_	**OR**
Test 1	>0.05	0.62	>0.05	1.54	>0.05	6.17
Test 2	>0.05	1.49	0.008	2.38	<0.0006	4.25
Test 3	<0.0006	5.40	<0.0006	8.10	0.0012	33.9
Test 4	<0.0006	0.17	>0.05	0.45	>0.05	0.77

^a^BD, Behçet’s disease; HLA, human leukocyte antigen; odds ratio; *P*_c_, corrected value of P. Test 1: ++ vs. –+ was performed to determine whether the HLA proposed factor was associated in B*51 positives. Test 2: +- vs. –– was designed to determine whether the HLA proposed factor was associated in B*51 negatives. Test 3: ++ vs. –– was conducted to determine the combined association between the HLA proposed factor and HLA-B*51. Test 4: +- vs. –+ was done to determine whether the associations between the HLA proposed factor and HLA-B*51 were different. *P*_c_ was calculated from the different 2 × 2 tables using Fisher’s exact test and corrected by multiplying by 6 (see [23]). Each comparison included B*51 and other HLA risk factors associated with the disease or suggestive of an association with the disease.

Next, we genotyped different genes described as associated with BD in different GWASs by selecting SNPs with MAF greater than 0.15 in the CEU population. The genotyping success ratio was greater than 96%, and the study population was found to be within the Hardy–Weinberg equilibrium for all the polymorphisms analysed (*P* > 0.05). The statistical power was 50% to 73% to detect an OR ≥1.3, depending on the MAF of each SNP. According to the results of univariate analysis (Table 4), the three SNPs located in *IL23R* and one of the SNPs within *IL10* were associated with BD in our study population. We analysed the best model for all the combinations from one to six attributes using the MDR system, including in the model all those HLA and non-HLA attributes suggestive of or associated with the disease as risk factors (B*51, B*57, A*02, A*24, rs17375018 and rs2222202). The best one-locus model, as expected, was HLA-B*51 (TA = 0.6556, CVC = 10/10; *P*_c_ < 0.0001), and the best two-attribute model was HLA-B*51+HLA-B*57, which had the highest TA (TA = 0.6845, CVC = 10/10; *P*_c_ < 0.0001).

**Table 4:** **One-locus analysis of the non–human leukocyte antigen genes included in this study**^**a**^

**SNPs**	**Location**	**BD patients**	**Controls**	***P***	**OR (95% CI)**
*LOC100129342*					
rs11206377	Chr1:39821691				
A		280 (0.48)	279 (0.45)		
G		306 (0.52)	347 (0.55)	>0.05	
*IL23R*					
rs17375018	Chr1: 67427735				
A		149 (0.26)	197 (0.32)		
G		431 (0.74)	415 (0.68)	0.01	1.37 (1.06-1.78)
rs7517847	Chr1:67454257				
G		189 (0.33)	272 (0.43)		
T		391 (0.67)	354 (0.57)	0.0001	1.59 (1.25-2.02)
rs1343151	Chr1:67491717				
A		192 (0.33)	250 (0.40)		
G		390 (0.67)	374 (0.60)	0.01	1.36 (1.07-1.73)
*IL10*					
rs3024498	Chr1: 205008152				
C		89 (0.15)	115 (0.18)		
T		495 (0.85)	507 (0.82)	>0.05	
rs2222202	Chr1:205012004				
A		200 (0.34)	254 (0.41)		
G		388 (0.66)	366 (0.59)	0.01	1.35 (1.06-1.71)
rs1800872	Chr1:205013030				
A		172 (0.30)	157 (0.25)		
C		408 (0.70)	463 (0.75)	>0.05	
*CPVL*					
rs317711	Chr7:29068833				
C		143 (0.25)	164 (0.27)		
G		429 (0.75)	454 (0.73)	>0.05	
*TDRD7*					
rs2061634	Chr9:99145603				
C		453 (0.79)	468 (0.75)		
G		123 (0.21)	154 (0.25)	>0.05	
*UBASH3B*					
rs4936742	Chr11:12214629				
C		302 (0.54)	327 (0.53)		
T		258 (0.46)	291 (0.47)	>0.05	
*UBAC2*					
rs7999348	Chr13:98730923				
A		407 (0.71)	430 (0.70)		
G		169 (0.29)	182 (0.30)	>0.05	

^a^BD, Behçet’s disease; CI, confidence interval; OR, odds ratio; *P*_c_, conditional probability; SNP, single-nucleotide polymorphism. An allelic model was used. Allelic frequencies are the number of each specific allele divided by the total number of alleles (number of individuals × 2) are shown within the parentheses.

## Discussion

The first finding of the present work is the confirmation of a second HLA-B allele group as a risk factor for BD, at least in European populations. Thus, in addition to B*51, we confirmed association of B*57 with BD in a model of logistic regression analysis, an independent association according to the analysis recommended by Svejgaard and Ryder [24] and that the model B*51+B*57 performed the best in the two-attribute model in the MDR analysis including HLA and non-HLA factors associated with the disease, Consistent with one previous study describing the same association in a UK cohort, the OR for B*57 was similar to that for B*51 in our study population [7]. Moreover, HLA-B*35 was detected as protective in our cohort. This HLA-B allele group had previously been found to be protective in an Italian cohort with a very limited sample size [28]. Interestingly, our study suggests a protective effect of B*58 because its frequency is significantly decreased among patients before correction and remains associated in the logistic regression analysis. Although this result needs confirmation, it is in agreement with different reports where a slight decrease in the frequency of HLA-B*58 was observed [6,8,28]. Both B57 and B58 have lower worldwide frequencies than B51 and B35; therefore, the statistical power to detect associations with the same ORs is worse. Noticeably, the only patient bearing B*58 had also B*51. HLA-B51 and B57 have the Bw4 epitope which is the ligand of the natural killer (NK) cell inhibitory receptor KIR3DL1. Interactions between Bw4 and KIR3DL1 have been proposed as a possible underlying mechanism to explain the relationship between HLA-B and BD. However, a large number of common HLA-B alleles encoding molecules with the Bw4 epitope (for example, B*44, B*49) have never been associated with BD. In this regard, B*35, protective according to our results, encodes molecules without the Bw4 epitope, but B*58 (which our study suggests is protective) encodes HLA molecules with this epitope. It has been described that changes at specific positions of the HLA-B molecule affect the interaction of Bw4 with KIR3DL1 [29]. Among them, position 97 (α_2_ domain) is very interesting because most of the HLA-B alleles with the Bw4 epitope (including B*58) encode proteins with 97R or 97S. The only exceptions are proteins encoded by alleles of the groups HLA-B*13, HLA-B*27, HLA-B*51, HLA-B*52 and HLA-B*57 [30]. From among these groups, B*51 and B*57 have been associated with BD, and HLA-B*27 was not found to be associated with the disease in our cohort. However, HLA-B*27 has been associated with BD in other studies [8,10]. The remaining groups (B*13 and B*52) have not been consistently associated with BD. HLA-B*51 and HLA-B*52 differ in only two amino acid positions, one of which is position 67 (α_1_ domain) (F67S). Changes in position 67 also affect the interaction of Bw4 with KIR3DL1 [29]. The amino acid in position 67 is shared between B*52 and B*13 [30]. Therefore, a possible explanation for the association of HLA-B and BD is that these molecules have, in addition to the Bw4 epitope, amino acid changes in specific positions such as 67 and 97, which modify interactions with KIR3DL1. Thus, determining which of the HLA-B alleles are involved in the disease could contribute to clarification in the etiology of BD.

The HLA-A region has been associated with the disease in GWASs, as well as in candidate gene studies, but it is not clear whether it is the primary associated locus. The distribution of the HLA-A groups was different in our patient and control cohorts. Nevertheless, in the univariate analysis, none of the differences reach the significance level necessary to be considered as associated, although three of them were suggestive of an association, two as risk factors and one as a protective factor. Moreover, HLA-A*24 was independently associated according to the logistic regression analysis and the analysis recommended by Svejgaard and Ryder [24]. Our results, as well as the previously published GWASs, suggest that the weight of the HLA-A region in the susceptibility to BD is weaker than that of the HLA-B region. Relatively few studies have investigated the influence of the HLA-A polymorphism in BD, and the associations described have not been consistent. HLA-A*26 has been described as associated with BD in Asian populations [5,16], but only one study in a Greek European population with a very limited number of patients reported an association with HLA-A26 [31]. We did not detect differences in the distribution of A*26 between patients and controls. Interestingly, A*24, a suggested risk factor in our work, is one of the few HLA-A specificities with the Bw4 epitope. Taking our data and those reported in other studies to date, we think that, contrary to the situation with HLA-B, a worldwide association of HLA-A with BD is unlikely to be found. Additionally, it is not completely clear whether other genes located near the HLA-A locus, such as HLA-E (ligand of the NK receptor CD94:NKG2A) could be primarily responsible for the association observed. In this way, HLA-E is highly selective in binding peptides derived from HLA class I leader sequences, and this condition regulates its expression on the cell surface [32]. Thus, polymorphisms located in the leader sequence could influence the expression of HLA-E in the cell surface and, as a consequence, the NK activity. Further studies are needed to clarify which HLA-A allele groups are associated in each ethnic group as well as the role of the HLA-A region in BD.

Regarding the bases of the HLA association with BD, a study in which three different peaks with independent effects were identified and replicated in the region was published while our manuscript was under review. That study also suggested that the association with the HLA-B*51 could be due to a noncoding variant located between HLA-B and MICA [13]. The results of the study by Hughes *et al*. are in agreement with the findings of other previously reported studies [33]. Nevertheless, studies designed to assess the association between MICA and BD have had conflicting results, although most of them support the association of MICA being due to linkage disequilibrium with HLA-B51. Moreover, different GWASs have not found an independent association between MICA and BD [16,19,20]. In the study by Hughes *et al*., HLA typing was deduced by imputation from SNPs. Imputation is a valuable tool for identifying and fine-mapping association between one region and one disease, as well as to extract much more information from existing GWAS data sets. Nevertheless, the accuracy of the imputation is not 100%, depending on the ethnicity of the population and the HLA locus under study [34]. Therefore, it would be interesting to compare these results where the HLA typing was assigned by imputation with those obtained by HLA typing to further clarify this question.

Regarding the non-HLA genes included in the study, *IL23R* is the most strongly associated in our population because the three SNPs within this region were associated with BD, with ORs ranging from 1.36 to 1.59. One of the SNPs located in *IL10* was also significantly associated. According to our sample size, we can detect associations with ORs of about 1.3. The SNPs rs11206377 (intergene), rs3024498 (*IL10*), rs1800872 (*IL10*) and rs2061634 (*TDR7*) had ORs between 1.1 and 1.3 in our cohort; therefore, our study is underpowered in these cases. The rest of the SNPs included do not seem to greatly influence susceptibility to BD in our population, because they have ORs lower than 1.1. Specifically, rs799348, a functional SNP in *UBAC2* which has been strongly associated with BD in the Turkish population, has an OR of 1.0 in our population. *IL23R* and *IL10* genes have been found to be associated to BD in GWASs as well as in candidate gene studies, and this association has been replicated in other populations [19,20,22,35]. Polymorphisms in *IL23R* have been associated with Crohn’s disease, rheumatoid arthritis and ankylosing spondylitis, and variants of *IL10* have been associated with ulcerative colitis and systemic lupus erythematosus. Therefore, these genes could be involved in a common pathway of susceptibility to several autoimmune and autoinflammatory diseases [36-38].

## Conclusion

Our study demonstrates an association of BD with B*57 independently of B*51, at least in European populations. Regarding HLA-A, taking our data together with those reported to date, a worldwide association of HLA-A with this disease is unlikely to be found. With respect to non-HLA genes, *IL-23R* and *IL10* played a role in this disease in our population.

## Abbreviations

BD: Behçet’s disease; CEU: Utah residents with Northern and Western European ancestry from the Centre d’Etude du Polymorphisme Humain collection; CVC: Cross-validation consistency; GWAS: Genomewide association study; HLA: Human leukocyte antigen; MAF: Minor allele frequency; MDR: Multifactor dimensionality reduction; OR: Odds ratio; SNP: Single nucleotide polymorphism; TA: Testing accuracy.

## Competing interests

The authors declare that they have no competing interests.

## Authors’ contributions

MAMC, JRGL and MFGE contributed to the conception and design of the research, data analysis and interpretation and drafting and revision of the manuscript. MCJ and LOF performed the experiments and analysed the data. NOC, MJCP, GE, GGG, MAGG, ACBM, RS, PF, TC, SC, JSB, ANR and JM contributed samples and patient information. All authors read and approved the final manuscript.
